# Specialty-Related Differences in Adherence to Antibiotic Therapy Guidelines for Infective Endocarditis in Older Patients

**DOI:** 10.3390/antibiotics15090837

**Published:** 2026-08-29

**Authors:** Lidvine Godaert, Claire Roubaud-Baudron, Emmanuel Forestier, Moustapha Dramé, the ENDOLA Study Group

**Affiliations:** 1 Department of Oncology, General Hospital of Valenciennes, 114 Avenue Désandrouin, 59300 Valenciennes, France; 2 EpiCliV Research Unit, Faculty of Medicine, University of the French West Indies, 97261 Fort-de-France, France; moustapha.drame@chu-martinique.fr; 3 Inserm BRIC UMR1312, University of Bordeaux, 33000 Bordeaux, France; claire.roubaud@chu-bordeaux.fr; 4 Pôle de Gérontologie Clinique, CHU Bordeaux, 33000 Bordeaux, France; 5 Department of Infectious Diseases, Centre Hospitalier Metropole Savoie, 73000 Chambéry, France; emmanuel.forestier@ch-metropole-savoie.fr; 6 Department of Clinical Research and Innovation, University Hospitals of Martinique, 97261 Fort-de-France, France

**Keywords:** infective endocarditis, older adults, antibiotic therapy, guidelines, medical ward

## Abstract

**Background/Objectives**: Infective endocarditis (IE) is a condition with complex management, and several guidelines exist to guide clinicians. Adherence to these recommendations varies and could even be described as poor. The aim of this study was to determine whether the medical specialty of the unit that initiated first-line treatment for IE was associated with risk factors of non-adherence to the 2015 ESC treatment guidelines in a population of patients aged 75 years and over. **Method**: We analysed data from patients with possible or definite IE included in the ENDOLA cohort, an observational, longitudinal, retrospective, nationwide, real-life cohort study involving 34 French hospitals. Logistic regression was performed using a manual stepwise method to identify variables independently associated with non-compliance of antibiotic treatment of IE with the recommendations of the 2015 ESC guidelines. **Results**: In total, 899 patients were included (Mean age: 83 ± 5 years, mean Charlson comorbidity score: 3 ± 2). The distribution across the medical units was 51.9% infectious disease (n = 467), 19.8% geriatric ward (n = 178), 18.5% cardiology (n = 166), and 9.8% other wards (n = 88). Patients admitted to geriatric wards were older (*p* < 0.0001) and were more likely to be dependent for activities of daily living (*p* < 0.0001). Admission to a geriatric unit was associated with a higher rate of non-compliance with the ESC guidelines (OR 1.6 (1.1–2.4); *p*: 0.03); prescriptions by cardiologists were more often in compliance with the guidelines (OR 0.6 (0.4–1.0); *p*: 0.04). **Conclusions**: The first-line antibiotic treatment prescribed for IE varies considerably depending on the unit responsible for providing care at the time of initial admission.

## 1. Introduction

Infective endocarditis (IE) is a condition whose incidence is increasing over time, particularly among older people [1,2,3,4]. Its management is complex, and several guidelines exist to guide clinicians [5,6,7]. IE is a difficult condition to treat, requiring long-term antibiotic therapy at an effective dose to minimise the risk of recurrence [6,7]. The guidelines provide recommendations regarding the recommended courses of curative antibiotic treatment, but patient outcomes depend, in part, on how quickly treatment is initiated. For this reason, guidelines recommend antibiotic regimens based on the identified pathogen, the type of valve involved (prosthetic or native) and any allergies the patient may have. The ESC guidelines [5] go beyond antibiotic treatment. In particular, they define the diagnostic criteria for infectious endocarditis, the place of endocarditis teams, and the indication for surgical management. A chapter is dedicated to adapted management in certain specific circumstances (pregnant women, congenital heart disease, etc.), but no adjustments are recommended specifically for older adults. Adherence to these recommendations varies, and in some cases could even be described as poor. Pallotto et al. [8] reported that, in an adult population with endocarditis, approximately one in two patients received first-line antibiotic treatment in line with the ESC 2015 guidelines. Tissot et al. [9] made a similar observation, showing that the guidelines were followed on average by 58% of the specialists who had developed them.

Older patients with IE are particularly challenging to treat, as treatment requires prolonged high-dose antibiotic therapy, which is usually administered intravenously. Older patients experience age-related changes to the immune system, resulting in greater susceptibility to severe sepsis [10]; they are often taking multiple medications and are more likely to have chronic kidney disease, which increases their risk of adverse drug reactions [11]. Despite these specific characteristics, there are no tailored recommendations for the antibiotic treatment of bacterial endocarditis in the older population. Godaert et al. [12] found in a cohort of older people that first-line antibiotic treatment recommendations were followed in 44% of cases, a compliance rate similar to that observed in a population of younger adults. However, the reasons for this lack of adherence are unknown. Some authors have found that compliance with the guidelines may vary according to the physician’s specialty, practice location or level of experience [13]. Identifying the factors associated with better adherence to the guidelines would likely help to improve their impact. Whilst adherence to the guidelines varies depending on the medical specialism responsible for initiating treatment, this point could be considered in optimising the care pathway for older patients with infective endocarditis.

The aim of this study was to determine whether the medical specialty of the unit that initiated first-line treatment for IE was associated with risk factor of non-adherence to the 2015 ESC treatment guidelines in a population of patients aged 75 years and over.

## 2. Results

**Figure 1:**
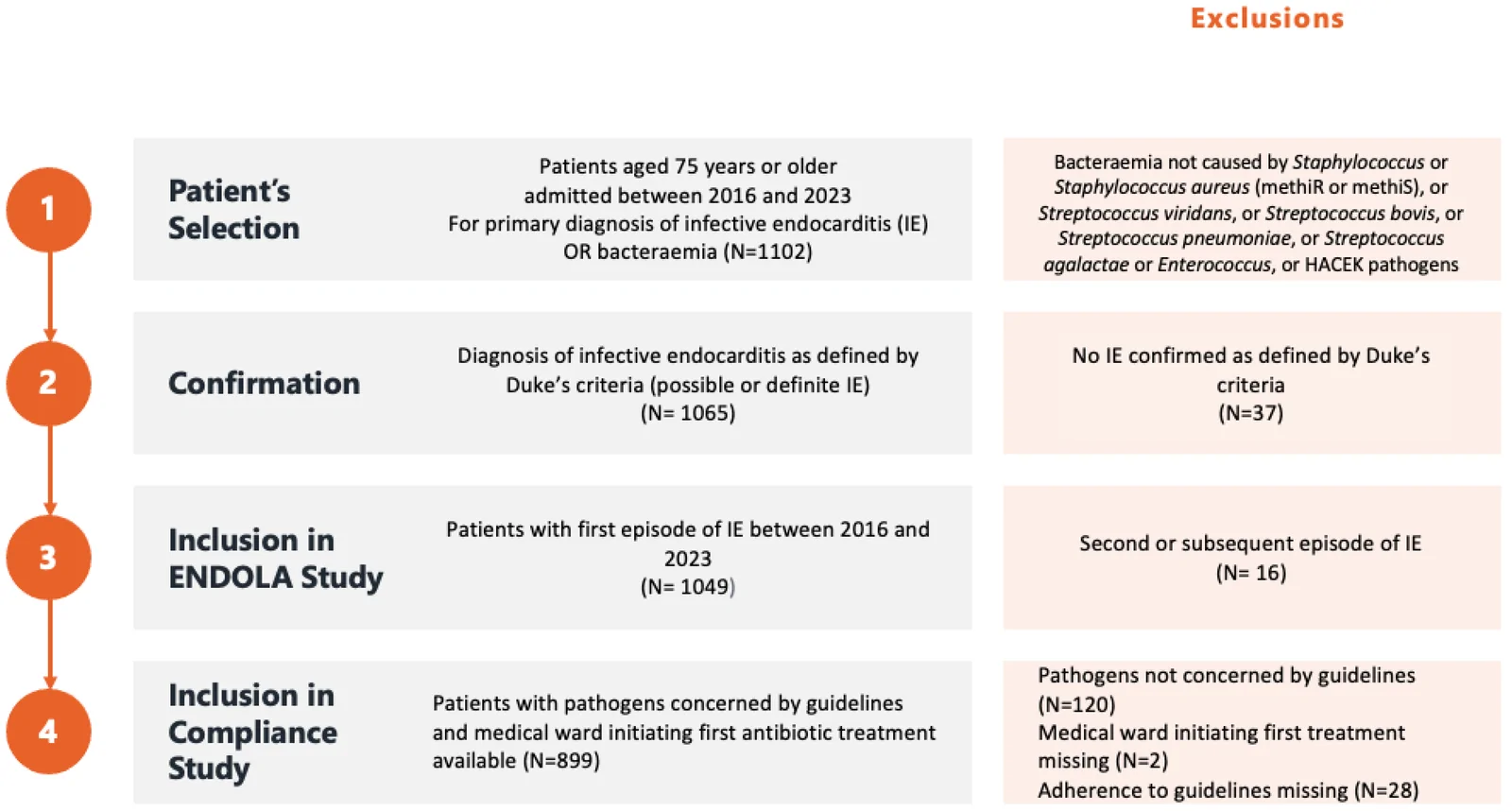
Flowchart of patients included in ENDOLA Study—compliance with guidelines according to first medical ward.

In total, 899 patients were included in the present study, with an average age of 83 ± 5 years, a mean Body Mass Index (BMI) of 26 ± 5 kg/m^2^, and a mean Charlson comorbidity score of 3 ± 2. The flowchart of patients included in the present analysis is shown in Figure 1. The demographic and clinical characteristics of the study population are shown in Table 1.

Patients admitted to geriatric wards were older (aged 85 years or older: 63.5% in the geriatric ward; 36.8% in the cardiology ward; 36.6% in the infectious diseases ward; 31.8% in others, *p* < 0.0001), were less likely to have been admitted from their home (86.5% versus 98.1% in the cardiology ward, 94.1% in the infectious diseases ward, 94.1% in others, *p* = 0.0003) and were more likely to be dependent for activities of daily living (ADL) (59.4% versus 41.9% in the cardiology ward, 39.7% in the infectious diseases ward, 36.3% in others, *p* < 0.0001). A surgical indication in the management of IE was less common (29.6% versus 57.8% in the cardiology ward, 43.1% in the infectious diseases ward, 46.0% in other wards *p* < 0.0001) and patients with a surgical indication were less likely to undergo surgery than those admitted to other wards (4.0% versus 28.7% in the cardiology ward, 15.5% in the infectious diseases ward, 9.2% in other wards *p* < 0.0001). The overall rate of non-adherence with the guidelines was 45.4%. Non-adherence rates were 51.7% in geriatric wards, 38.6% in cardiology wards, and 43.3% in infectious diseases wards.

Table 2 details the factors associated with the risk of non-compliance with the 2015 ESC guidelines in terms of first-line antibiotic treatment for IE. Admission to a geriatric unit and initiation of the first-line antibiotic treatment in geriatrics were associated with a higher rate of non-compliance with the ESC guidelines [5]. Of the 929 patients initially included, 463 were included in the various models because of missing data for some explanatory variables. Nevertheless, according to the Hosmer–Lemeshow test, all models showed a very good fit to the data, with p-values of 0.92 for Model 1, 0.76 for Model 2, 0.96 for Model 3, and 0.97 for Model 4.

## 3. Discussion

In our study, being initially admitted to a geriatric unit, with initiation in geriatrics of first-line antibiotic treatment for IE, was associated with a greater risk of non-adherence to the 2015 ESC guidelines [5] (OR = 1.6 (1.1–2.4); *p* = 0.03). Conversely, initial admission to a cardiology unit, with initiation of first-line antibiotic treatment for IE by the cardiologists, was associated with greater adherence to the 2015 ESC guidelines [5] (OR = 0.6 (0.4–1.0); *p* = 0.04). Admission to an infectious diseases unit was not associated with adherence to the treatment guidelines in our analysis. In our cohort, patients admitted to a geriatric unit were older and more dependent, and therefore frailer. These factors expose them to a greater risk of adverse events of treatment (such as confusion, renal failure, etc.) [14,15,16]. These characteristics imply that they may be less suitable for first-line treatment for IE, which is not adapted to each patient in terms of the specific drug or route of administration [14,15,16]. The adaptation of treatments by geriatricians to patients’ frailty profiles is common practice in this medical discipline. Older adults are under-represented in studies, as underlined by Markham et al. [17]. This reality forces geriatricians to personalize treatments according to their patients’ risk profiles. Patients’ clinical profiles (age, frailty, etc.) may influence adherence to treatment recommendations [18]. In frail or very ill geriatric patients, geriatricians sometimes opt for a first-line treatment considered to be less risky for the patient, and then intensify treatment at a later stage if the patient’s clinical condition improves. Furthermore, the atypical presentation of many diseases in geriatric patients can result in a longer time to diagnosis [19]. In our study, only the first prescribed course of treatment was taken into account when analysing compliance with the guidelines for first-line treatment of IE. It is also possible that geriatricians are less familiar with the guidelines than other specialists who are usually responsible for treating infective endocarditis. This justifies the intervention of an endocarditis team (ET) in the management of these patients, where available. Alternatively, a consultation with an infectious diseases specialist should be requested.

Another hypothesis is that cardiologists may be less inclined to diverge from clinical guidelines, and more particularly because they may be less experienced at adapting antibiotic treatments to the frailty profile of older patients. According to the results of our bivariate analysis, endocarditis teams (ET) were more frequently involved in the management of patients with IE who were admitted to cardiology wards, as compared to patients admitted to other care units (with involvement of a multidisciplinary endocarditis team in 59.4% of patients in cardiology wards versus 30.1% in geriatric wards, 32.8% in infectious disease wards, 29.9% in others, *p* < 0.0001). The decision regarding the care plan is then made collectively, as outlined in the recommendations [5,6]. The proposed composition of the ET [6] encompasses core members, including a cardiologist, cardiac imaging expert, cardiovascular surgeon, infectious disease specialist, microbiologist, and specialist in outpatient parenteral antibiotic treatment. Geriatricians are proposed as adjunct specialists. This may partly explain why recommendations are applied more strictly when they fail to take into account the physiological and metabolic characteristics of older or very old people, as these specificities are often not well understood in these specialties. When it comes to choosing between conflicting strategies found in different guidelines, Beraud et al. [13] showed the influence of medical specialty on IE treatment strategies. These authors also highlighted the influence of professional experience on treatment decisions. The factors influencing practitioners’ therapeutic decisions are numerous and difficult to list exhaustively. The collective consideration of all these complex factors at the patient’s bedside probably explains the moderate adherence of doctors to treatment recommendations, even in the case of physicians who were themselves involved in drafting the recommendations in question [9]. Depending on their original medical specialty or experience, doctors may be more or less willing to make such adjustments.

It is difficult to determine what impact adherence to guidelines on first-line antibiotic treatment for IE has on patient outcomes, particularly in older patients. Yet this is probably the only question of interest. If we assume that the guidelines improve patient outcomes, then adherence to them is of paramount importance. However, what may be true at a population level (i.e., what will prove true across a large cohort of patients) is not necessarily true at an individual level [19]. Indeed, there is considerable heterogeneity in individual factors within a geriatric population, as each person ages in a unique way, influenced by the diseases they encounter, life events, lifestyle habits, or the environment in which they live (climatic, health-related, social, emotional, etc.). As the management of IE is complex and often involves both medical and surgical approaches, it is difficult to assess the specific contribution of first-line antibiotic therapy to long-term outcomes independently. Some older patients may be excluded from surgical treatment for several reasons (e.g., risk considered too high, refusal by the patient or their family, lack of available care in the receiving facility, or patients considered too frail for transfer, etc.) [20,21,22,23]. In our study, patients with IE admitted to geriatric wards were less likely to have an indication for surgical treatment than those admitted to other wards (29.6% versus 57.8% in the cardiology ward, 43.1% in the infectious diseases ward, 46.0% in other wards, *p* < 0.0001). One possible explanation is that the lower use of TOE in geriatric wards may have led to an underdiagnosis of lesions requiring surgical intervention. Additionally, among patients with surgical indication, those admitted to geriatrics underwent surgery less frequently than patients admitted to other wards (4.0% versus 28.7% in cardiology, 15.5% in infectious diseases, 9.2% in other wards, *p* < 0.0001). Patients with surgical indication who are managed exclusively with medical therapy have the poorest prognosis. With the surgical option excluded, medical management then becomes the main alternative, whereas this would not have been the case in theory. In this regard, it is well established that access to surgical management has a major impact on outcomes, even in older patients [24,25,26,27,28].

Several strengths of this study should be underlined. Firstly, it addressed a central and underexplored problem, namely the influence of the admitting specialty on adherence to treatment guidelines in older IE patients. Secondly, the interpretation of the results takes into account the complexity of geriatric care, particularly frailty, comorbidities, and individualized therapeutic decision-making. Finally, the potential role of multidisciplinary endocarditis teams in improving guideline adherence was also discussed. Although involvement of a multidisciplinary endocarditis team may contribute to improved adherence to guideline recommendations, this variable was not retained in the multivariable models, in accordance with our predefined variable-selection strategy. Its associated p-value was 0.201, exceeding the prespecified exclusion threshold of *p* < 0.10. Therefore, including this variable in the final models would have required a post hoc deviation from the predefined analytical approach. However, some limitations deserve to be acknowledged. Because of its observational design, causal relationships cannot be established. Differences exist in baseline characteristics between patients admitted to geriatric and cardiology wards, specifically regarding frailty and comorbidity, which may have introduced confusion bias. However, multivariable analysis helps to reduce confounding effects. A second limitation is that only the initial antibiotic regimen was analyzed, without considering subsequent therapeutic adaptations. Another limitation is the substantial amount of missing data for several explanatory variables, which reduced the number of patients available for multivariable analyses from 929 to 463. The use of complete-case analyses may have reduced statistical power and introduced selection bias, and the results should therefore be interpreted with caution. The relatively limited and uneven number of patients across individual years precluded a robust assessment of temporal trends in adherence over the 2016–2023 study period. Consequently, changes in adherence over time could not be reliably evaluated. Finally, the study did not assess the impact of adherence to guidelines on clinical outcomes such as mortality or quality of life, and some deviations from the guidelines may have been based on by clinically justified adaptations in frail older patients. IE management is highly complex and relies on multiple interacting factors, including surgical eligibility, comorbidities, frailty, and multidisciplinary decision-making. Therefore, isolating the specific impact of first-line antibiotic guideline adherence on outcomes remains challenging in this population.

Despite the existence of clinical guidelines, some of which are very recent, the first-line antibiotic treatment prescribed for IE varies considerably depending on the unit responsible for providing care at the time of initial admission. The frailty profile of patients with IE appears to determine which medical unit they are referred to for management. In our study, prescriptions by cardiologists were more often in compliance with the guidelines. It seems particularly important to examine the impact of these different courses of treatment on patient outcomes in terms of both survival and quality of life.

## 4. Materials and Methods

### 4.1. Study Design and Study Population

**Figure 2:**
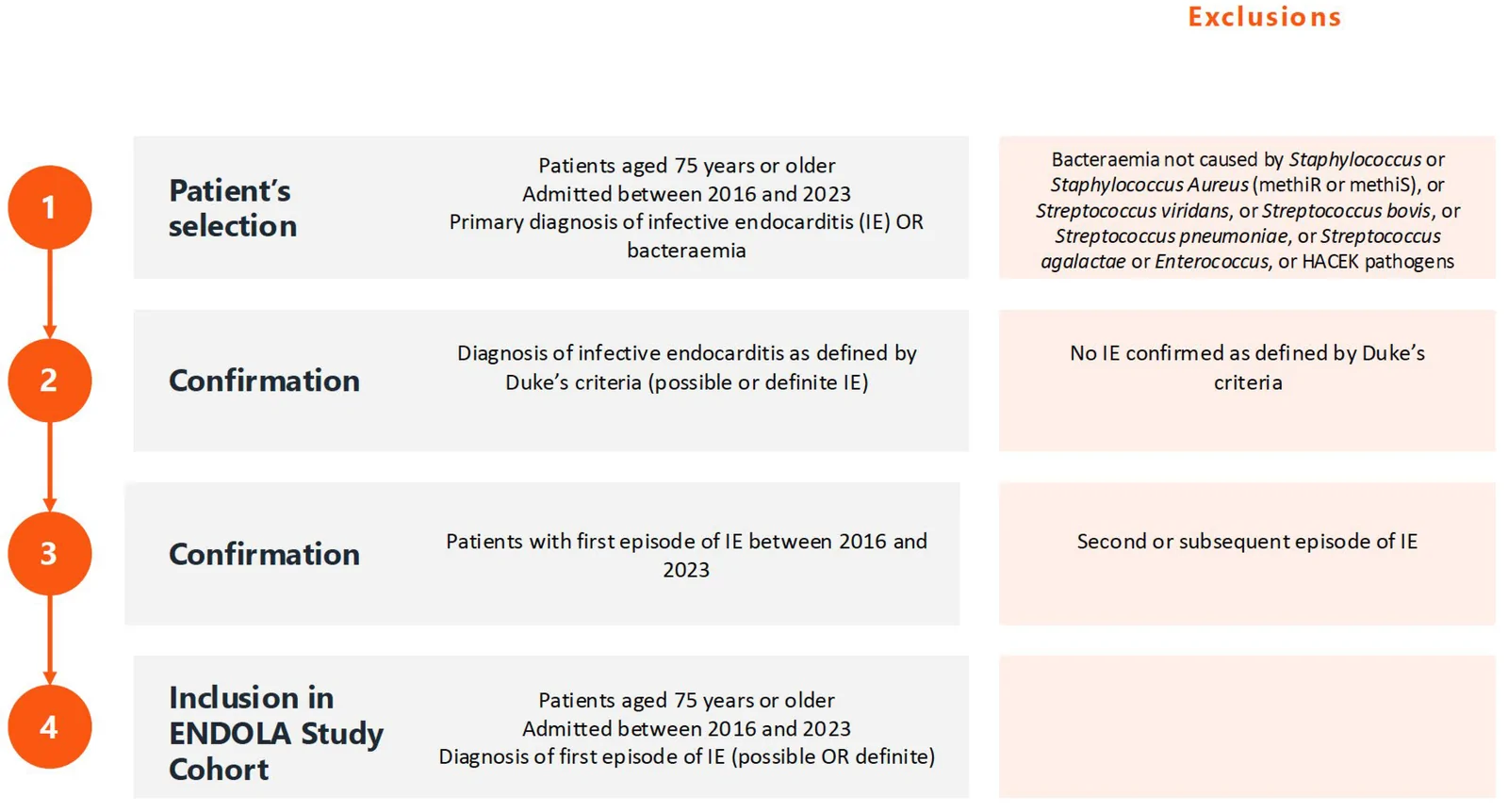
Algorithm for selecting patients included in ENDOLA Study.

The ENDOLA study was an observational, longitudinal, retrospective, nationwide, real-life cohort study involving 34 French hospitals. To be included in ENDOLA Study, patients had to be aged 75 years or older, admitted to one of the participating centres between 2016 and 2023, with a primary diagnosis of possible or definite IE (defined by Duke’s criteria). The algorithm for patient selection in the ENDOLA study is described in Figure 2. Patients were excluded from the study if they explicitly objected to the use of their medical files for research purposes.

For the present analysis, we considered only patients included in the ENDOLA cohort who had IE caused by pathogens for which the 2015 ESC guidelines provide treatment recommendations and for whom the unit that initiated treatment was known.

### 4.2. Study Data

The following data were retrieved for all patients from the medical records: demographic characteristics (age, sex, place of residence), use of a home nurse, clinical data such as body mass index (BMI), dependence as assessed by Katz’s activities of daily living [29] (a patient was considered dependent if unable to perform at least one task without help), presence of allergy to antibiotics, level of comorbidity according to Charlson’s comorbidity index [30], condition of heart valves, presence of implantable cardiac device (i.e., pacemaker or defibrillator), and history of IE. On hospital admission for the index acute episode and/or during the index stay, the following variables were recorded: ward initiating first-line antibiotic treatment after identification of the pathogen responsible for the IE (geriatrics ward, cardiology ward, infectious diseases ward, other wards), presence of fever, presence of clinical signs/symptoms of cardiac decompensation, presumed origin of IE, type of valves (native or prosthetic), presence of vegetations, associated valve damage, presence of neurological signs/symptoms, renal failure at admission, arthritis, results of transthoracic/trans-oesophageal echography (TTE/TOE), results of positron emission tomography (PET) scan. We also recorded whether an endocarditis team defined by ESC guidelines [6] was involved in the patient’s management or not. Antibiotic treatment of IE was recorded by identifying each line of treatment.

Compliance with guidelines regarding antibiotic therapy was defined according to the 2015 guidelines of the European Society of Cardiology (ESC) for the management of IE, which were in force at the time of the study [5]. Recruitment for the cohort took place between 2016 and 2023. ESC guidelines published in 2023 were not being followed at the participating centers and therefore could not be used for this study. Accordingly, patients were categorized according to whether or not their first-line antibiotic prescription was compliant with the ESC 2015 guidelines, taking account of the pathogen identified, the presence or absence of a prosthetic valve, and the presence or absence of allergy. Dosage and route of administration of the antibiotics were not considered in evaluating compliance with the guidelines. Three medical wards were analysed, namely infectious diseases, cardiology, and geriatrics.

### 4.3. Statistical Analyses

Continuous variables are described as mean and standard deviation or median and range, as appropriate. Categorical variables are described as number and percentage. Bivariable analyses were performed using logistic regression with one explanatory variable. In multivariable analysis, logistic regression was performed using a manual stepwise method to identify variables independently associated with non-compliance of antibiotic treatment of IE with the recommendations of the 2015 ESC guidelines [5]. The threshold probability for entering variables into model 1 was *p* < 0.20. Removal threshold was *p* < 0.1. Initially, we identified the factors independently associated with non-adherence to guidelines for IE antibiotic treatment, without accounting for the admitting department (Model 1). These variables were then used systematically for adjustment in subsequent models, with the geriatric department added in Model 2, the cardiology department in Model 3, and the infectious diseases department in Model 4. Model goodness-of-fit was tested using the Hosmer and Lemeshow test. Tests were considered significant for *p*-values < 0.05. Statistical analyses were performed using SAS version 9.4 (SAS Institute Inc., Cary, NC, USA).

## Figures and Tables

**Table 1:** Demographic, clinical, and microbiological characteristics of older adults with infective endocarditis, by clinical ward (n = 899).

	Geriatrics		Cardiology		Infectious Diseases		Others		*p*
Characteristics	n	%	n	%	n	%	n	%	
Age 85 years or older	113	63.5	61	36.8	171	36.6	28	31.8	<0.0001
Female sex	88	49.4	58	34.9	170	36.4	40	45.5	0.008
Usual place of residence = Home	154	86.5	156	98.1	431	94.1	80	94.1	0.0003
Dependant for ADLs	104	59.4	67	41.9	176	39.7	29	36.5	<0.0001
Presence of comorbidity	115	64.6	105	63.3	314	67.2	63	71.6	0.53
BMI < 18.5 kg/m^2^	8	5.5	4	2.6	20	4.7	3	4.0	0.64
Baseline cardiac status									
Previous infective endocarditis	7	4.0	10	6.0	39	8.4	6	6.9	0.25
Natural aortic valve	132	74.2	99	59.6	282	60.4	60	69.0	0.005
Natural mitral valve	167	94.4	155	93.4	421	90.5	79	91.9	0.38
Natural tricuspid valve	176	99.4	163	98.2	457	98.5	86	100.0	0.48
Natural pulmonary valve	177	100.0	166	100.0	465	100.0	86	100.0	
Presence of pacemaker or defibrillator	40	22.6	52	31.3	113	24.3	16	18.2	0.09
Symptoms at admission									
Fever	138	78.9	119	75.3	369	80.9	68	77.3	0.48
Heart failure	85	48.9	77	46.7	161	34.9	32	36.4	0.003
Neurologic symptoms	30	17.2	41	25.5	108	23.8	41	47.1	<0.0001
Renal failure at admission	60	34.1	63	38.4	175	37.9	40	47.1	0.25
Acute renal failure with haemodialysis	1	0.6	4	2.4	14	3.0	6	6.8	0.03
Arthritis	24	13.5	15	9.0	67	14.4	6	6.8	0.11
Suspected healthcare-associated endocarditis	41	24.3	31	20.4	64	15.8	13	19.7	0.12
Penicillin allergy	9	5.1	9	5.5	19	4.1	4	4.6	0.88
Involvement of a multidisciplinary endocarditis team	53	30.1	98	59.4	151	32.8	26	29.9	<0.0001
TTE performed	161	92.0	148	98.0	422	93.2	77	89.5	0.04
TOE performed	69	40.4	101	68.7	302	69.0	56	65.1	<0.0001
PET-CT scan performed	61	36.8	45	33.3	163	41.1	20	23.8	0.02
Presence of vegetation	94	58.0	108	72.5	291	64.0	58	69.9	0.04
Presence of valve damage	33	30.8	59	39.6	185	42.2	32	41.5	0.19
Surgical indication	52	29.6	96	57.8	199	43.1	40	46.0	<0.0001
Patient underwent surgery	7	4.0	47	28.7	67	15.5	8	9.2	<0.0001

Missing data: usual place of residence (n = 19); dependant for activities of daily living (ADLs) (n = 41); BMI (n = 100); previous infective endocarditis (n = 7); natural aortic valve (n = 1); natural mitral valve (n = 5); natural tricuspid valve (n = 6); natural pulmonary valve (n = 5); presence of pacemaker or defibrillator (n = 3); fever (n = 22); heart failure (n = 10); neurologic symptoms (n = 23); renal failure at admission (n = 12); suspected healthcare-associated endocarditis (n = 108); penicillin allergy (n = 1); intervention of a multidisciplinary endocarditis team (n = 11); Transthoracic Echocardiography (TTE) performed (n = 34); transoesophageal echocardiography (TOE) (n = 57); presence of vegetation (n = 50); presence of valve damage (n = 128); surgical indication (n = 8); patient operated on (n = 40). MSSA: Methy-sensible staphalycoccus aureus; MRSA: Methy-resistant staphalycoccus aureus.

**Table 2:** Multivariable analysis using logistic regression of the association between the clinical ward (geriatric ward, cardiologic ward, infectious disease ward) and non-compliance with the ESC guidelines for first-line antibiotic treatment in older adults with infective endocarditis.

	Model 1			Model 2			Model 3			Model 4		
Characteristic	OR	95% CI	*p*	OR	95% CI	*p*	OR	95% CI	*p*	OR	95% CI	*p*
BMI ≥ 25.0 kg/m^2^	1.6	1.1–2.4	0.01	1.7	1.1–2.5	0.01	1.7	1.1–2.5	0.01	1.7	1.1–2.5	0.01
Natural aortic valve	0.6	0.4–0.9	0.03	0.7	0.4–1.0	0.04	0.7	0.4–1.0	0.04	0.7	0.5–1.0	0.05
Natural mitral valve	0.3	0.2–0.7	0.004	0.4	0.2–0.8	0.009	0.4	0.2–0.8	0.009	0.4	0.2–0.8	0.008
Neurologic symptoms	1.5	1.0–2.3	0.07	1.4	0.9–2.2	0.11	1.5	0.9–2.3	0.09	1.4	0.9–2.2	0.13
Acute renal failure with haemodialysis	3.1	0.9–10.8	0.07	2.1	0.6–6.9	0.23	2.0	0.6–6.6	0.27	2.0	0.6–6.7	0.24
Suspected healthcare-associated endocarditis	3.0	1.8–4.9	<0.0001	2.7	1.6–4.5	<0.0001	2.9	1.7–4.8	<0.0001	2.7	1.7–4.5	<0.0001
Geriatric ward				1.6	1.1–2.4	0.03						
Cardiology ward							0.6	0.4–1.0	0.04			
Infectious diseases ward										1.1	0.7–1.6	0.72

Model 1: Model without comparison between wards. Model 2: Model 1 + comparison between geriatric ward and other wards (cardiology, infectious diseases and others). Model 3: Model 1 + comparison between cardiology ward and other wards (geriatrics, infectious diseases and others). Model 4: Model 1 + comparison between infectious diseases ward and other wards (geriatrics, cardiology and others).

## Data Availability

The data presented in this study are available on request due to French Laws regarding biomedical research on human beings, Minimal dataset cannot be made freely accessible.

## Abbreviations

The following abbreviations are used in this manuscript:

IE	Infective Endocarditis
ESC	European Society of Cardiology
BMI	Body Mass Index
ADL	Activities daily living
TTE	Transthoracic Echocardiography
TOE	Transoesophageal echocardiography
ET	Endocarditis teams
PET	Positron Emission Tomography
